# Development of new host‐specific *Bacteroides* qPCRs for the identification of fecal contamination sources in water

**DOI:** 10.1002/mbo3.313

**Published:** 2016-01-14

**Authors:** Marta Gómez‐Doñate, Arnau Casanovas‐Massana, Maite Muniesa, Anicet R. Blanch

**Affiliations:** ^1^ Department of MicrobiologyUniversity of BarcelonaDiagonal 643BarcelonaCatalonia08028Spain; ^2^ Department of Epidemiology of Microbial DiseasesYale School of Public HealthNew HavenConnecticut

**Keywords:** *Bacteroides*, DGGE, microbial source tracking, quantitative PCR

## Abstract

*Bacteroides* spp. have been proposed as indicators of fecal contamination in microbial source tracking (MST) methodologies. The aim of this study was to develop new qPCR assays that target host‐specific Bacteroidal 16S ribosomal RNA genes, to determine the source of fecal contamination in water. Denaturing gradient gel electrophoresis (DGGE) was used to select for host‐specific bands of *Bacteroides* associated with a fecal pollution source and later to design four qPCR host‐specific assays. A set of common primers for *Bacteroides* spp., four different *Bacteroides* spp. host‐associated hydrolysis probes (human, cattle, pig, and poultry), and one hydrolysis probe for the *Bacteroides* genus were designed. This set of qPCR assays together with other previously developed Bacteroidetes MST targets were used to analyze water samples with fecal contamination from the four sources studied. The host‐specific *Bacteroides* qPCRs designed for human (HMprobeBac), pig (PGprobeBac), and poultry (PLprobeBac) were highly specific for its sources (1.0, 0.97, and 1.0, respectively) although its sensitivity was lower (0.45, 0.50, and 0.73, respectively). The cattle‐specific qPCR was totally unspecific and was discarded for future experiments. When compared to previously designed assays, the human and pig qPCRs showed better accuracies (0.86 and 0.84) than their counterparts HF183 and Pig‐2‐Bac (0.38 and 0.65). Thus, the newly designed human, pig, and poultry qPCR assays outperform other methods developed until date and may be useful for source tracking purposes.

## Introduction

Fecal pollution of water is one the main causes of disease worldwide, particularly in developing countries (WHO 2008), and thus it is a global public health concern. In addition, it has a negative impact on many economic activities and leads to environmental deterioration. As a result, it is necessary to devise methods to monitor the levels of fecal pollution and determine its source(s) (Hagedorn et al. 2003). The identification and tracking of fecal source(s) may contribute to improving the management of the pollution at its source, thereby reducing economic losses and resolving legal issues (Jagals and Grabow 1996; Blanch et al. 2004).

Several microbiological methods such as those involving fecal coliforms, *Escherichia coli*, enterococci, and sulfite‐reducing clostridia are routinely used as microbial indicators to detect the presence of fecal pollution in water (Scott et al. 2002; Blanch et al. 2006). Unfortunately, such methods do not discriminate between human and animal fecal sources. In contrast, some *Bacteroides* spp. have been reported to be highly host specific (Kreader 1995) and consequently some species or markers have been proposed as new indicators of fecal pollution in water through microbial source tracking (MST) (Fiksdal et al. 1985; US EPA 2005). *Bacteroides* spp. are one of the most abundant genera of bacteria in the gastrointestinal tract of humans and other warm‐blooded animals. They represent up to a third of total gut microbiota (Eckburg et al. 2005; Ley et al. 2008; Wei et al. 2013; Ziemer 2014). Since *Bacteroides* spp. are anaerobic bacteria, cultivable *Bacteroides* can only survive 6 days under oxygen stress conditions and 2 or 3 days in the summer, with high temperatures (Avelar et al. 1998; Ballesté and Blanch 2010). This short environmental persistence hampers detection of *Bacteroides* by culture methods; however, their nucleic acids may persist longer periods (Kreader 1998).

For this reason, different MST molecular markers that target the order Bacteroidales have been proposed in recent years (Harwood et al. 2014). Among these, human‐associated HF183 (Bernhard and Field 2000a), belonging to *Bacteroides dorei* (Haugland et al. 2010), has been widely evaluated. However, it has been shown that HF183 is not entirely specific to human fecal sources (Green et al. 2014).

The aim of this study is to develop new host‐specific fecal pollution markers based on *Bacteroides* spp. To this end, the 16S rRNA genes of populations of *Bacteroides* occurring in samples with a unique and known fecal pollution source (cattle, pig, poultry, or human) were analyzed by denaturing gradient gel electrophoresis (DGGE). Host‐specific 16S rRNA gene sequences were selected to establish a set of common primers and various host‐specific hydrolysis probes that could be used in qPCR assays. The new qPCRs were then tested in point‐source water samples and compared with previously proposed MST molecular markers based on Bacteroidales.

## Materials and Methods

### Bacterial strains and growth conditions

*Bacteriodes coprophilus* DSM 18228^T^, *B. coprosuis* DSM 18011^T^, *B. eggerthii* DSM 20697^T^, *B. finegoldi* DSM 17565^T^, *B. fragilis* DSM 2151^T^, *B. gallinarum* DSM 18171^T^, *B. intestinalis* DSM 17393^T^, *B. ovatus* DSM 1896^T^, *B. pyogenes* DSM 20611^T^, *B. salanitronis* DSM 18170^T^, *B. suis* DSM 20612^T^, *B. thetaiotaomicron* DSM 2079^T^, *B. uniformis* DSM 6597^T^, *B. vulgatus* DSM 1447^T^, and *Parabacteroides distasonis* DSM 20701^T^ were used as reference strains to establish the optimal DGGE gradient and conditions.

Type strains were grown using BPRM (*Bacteroides* Phage Recovery Media) broth (Tartera et al. 1992) and BPRM agar (Pronadisa, Laboratorios Conda, Madrid, Spain) without antibiotics at 37°C in anaerobic conditions (Anaerocult^®^; Merck Millipore, Darmstadt, Germany).

### Host‐specific wastewater and slurry samples

To devise the qPCR assays, a total of 114 wastewater samples were collected from various sources and the DGGE assay was performed on them. Thirty‐four human sewage samples were obtained from five urban wastewater treatment plants located in the metropolitan area of Barcelona, Catalonia (NE Spain). Two of the wastewater treatment plants served populations of 384,000 inhabitants (28 samples); two plants served populations of between 100,000 and 200,000 inhabitants (four samples); and the other plant served a population of more than 2,000,000 inhabitants (two samples). All sewage samples were collected from the influent sewer entering the plants right after the bar screen and before the primary treatment.

A total of 25 samples of poultry slurry were collected from two poultry slaughterhouses that weekly process 60,000 chickens each. A total of 30 samples of pig slurry were obtained: 19 from four pig slaughterhouses that process 12,500, 15,000, 15,000, and 5000 pigs each week, respectively; and 11 from a fattening farm containing 14,000 pigs. Finally, 25 samples of cattle slurry were collected from six slaughterhouses processing between 250 and 2000 calves weekly (15 samples); and from two farms: one with 50 cows and the other with 200 calves (10 samples). All slaughterhouses were located in Catalonia (NE Spain) and had separate pipes for animal slurry and human wastewater from workers’ lavatories and showers.

In all samplings, 1 L of water or slurry were collected in sterile polyethylene containers and refrigerated at 4°C for up to 6 h before being processed in accordance with standardized protocols (Anonymous 1994).

Once the qPCR assays were designed and in order to test them, a total of 44 additional wastewater samples (11 human samples, 11 poultry samples, 12 pig samples, and 10 cattle samples) were freshly collected from the same locations described above.

### Enumeration of *Escherichia coli* and somatic coliphages

*Escherichia coli* and somatic coliphages were used as indicators of bacterial fecal pollution and viral fecal pollution, respectively. *Escherichia coli* was enumerated by membrane filtration, following previously standardized methods (American Public Health Association, American Water Works Association & Water Environment Federation 1999) using Chromocult^®^ Coliform agar (Merck, Darmstadt, Germany) incubated at 44.5°C for 24 h. Somatic coliphages were counted by the ISO 10705‐2 double agar layer method (Anonymous 2000) using *E. coli* strain WG5 (ATCC 700078) as the host. Each enumeration was performed in duplicate.

### Nucleic acid isolation

Genomic DNA was obtained from reference strains by centrifuging 1.5 mL of a 48 h culture at 12,000*g* for 5 min. The pellet was washed twice in Tris‐EDTA buffer (Tris‐HCl 10 mmol/L, pH = 8.0 and EDTA 1 mmol/L) and the cells were then incubated at 100°C for 10 min. Finally, the mixture was centrifuged at 12,000*g* for 5 min and the supernatant was subjected to DNA amplification.

DNA extraction from the samples was performed with the QIAamp DNA blood minikit (Qiagen GmbH, Hilden, Germany), following the manufacturer's instructions. The DNA was suspended in a final volume of 50 *μ*L of elution buffer. The integrity of the genomic DNA extracted was evaluated by 0.8% agarose gel electrophoresis and ethidium bromide staining.

### Primers and PCR amplification

The 32F and 708R primer pair (Table 1) was used to amplify a 670‐bp fragment of the 16S rRNA gene using the DNA extracted from each wastewater sample or from 2‐day‐old cultures of the reference strains. Each 25 *μ*L of PCR mixture contained 12.5 *μ*L 2X DreamTaq Green DNA Polymerase (Fermentas, Madrid, Spain), 400 nmol/L of each primer, and 2.5 *μ*L of DNA. PCR amplification was performed using a GeneAmp PCR system 2700 (Applied Biosystems, Barcelona, Spain) with the following conditions: initial denaturation step at 94°C for 5 min; then 35 cycles consisting of 94°C for 30 sec, 65°C for 1 min (this temperature was decreased by 1°C every cycle until the touchdown temperature of 61°C was reached), and 72°C for 1 min and 30 sec; and finally a 6 min extension at 72°C. *Bacteriodes fragilis* DSM 2151^T^ was used as a positive control; a negative control containing no template was also included in each experiment.

**Table 1:** Primers and probes used in this study

Name	Sequence (5′ to 3′)	Use	Target^a^	Reference
32F	AACGCTAGCTACAGGCTT	PCR and sequencing	*Bacteroides* genus specific	Bernhard and Field (2000b,b)
708R	CAATCGGAGTTCTTCGTG	PCR	*Bacteroides* genus specific	Bernhard and Field (2000b,b)
32F‐GC^b^	*CGCCCGGGGCGCGCCCCGGGCGGGGCGGGGGCACGGGGGG*AACGCTAGCTACAGGCTT	PCR‐DGGE	*Bacteroides* genus specific	Myers et al. (1985a,b)
580R	CGCTCCCTTTAAACCCAAT	DGGE‐PCR and sequencing	*Bacteroides* genus specific	This study
pGEMup	tgtaatacgactcactat	PCR and sequencing	pGEM‐T Easy plasmid	Serra‐Moreno et al. (2008)
Bac‐FW	GGCGCACGGGTGAGTAAC	qPCR	Uncultured *Bacteroides*	This study
Bac‐Rev	TGTGGGGGACCTTCCTCTC	qPCR	Uncultured *Bacteroides*	This study
HMprobeBac	FAM‐GTGAGGGCATCTAATCA	qPCR probe humans	Uncultured *Bacteroides*	This study
PLprobeBac	FAM‐TCCGCATGAAGGACTT	qPCR probe poultry	Uncultured *Bacteroides*	This study
PGprobeBac	FAM‐TATGATAGCATTAGAGTGTGACGAA	qPCR probe pigs	Uncultured *Bacteroides*	This study
CWprobeBac	FAM‐CTATGGGATGGGGATGC	qPCR probe cattle	Uncultured *Bacteroides*	This study
Bacprobe	FAM‐CGGGGTAACGGCCCA	qPCR common probe	*Bacteroides* genus specific	This study
HF183f	ATCATGAGTTCACATGTCCG	qPCR primers	Human *Bacteroides*	Seurinck et al. (2005)
HF183r	TACCCCGCCTACTATCTAATG	qPCR primers	Human *Bacteroides*	Seurinck et al. (2005)
Pig‐2‐Bac	FAM‐TCCACGGGATAGCC	qPCR probe pigs	Pig *Bacteroides*	Mieszkin et al. (2009)
Pig‐2‐Bac41F	GCATGAATTTAGCTTGCTAAATTTGAT	qPCR primers pigs	Pig *Bacteroides*	Mieszkin et al. (2009)
Pig‐2‐Bac163Rm	ACCTCATACGGTATTAATCCGC	qPCR primers pigs	Pig *Bacteroides*	Mieszkin et al. (2009)
Rum‐2‐Bac	FAM‐ATGAGGTGGATGGAATT	qPCR probe cattle	Cattle *Bacteroides*	Mieszkin et al. (2010)
BacB2‐590F	ACAGCCCGCGATTGATACTGGTAA	qPCR primers cattle	Cattle *Bacteroides*	Mieszkin et al. (2010)
Bac708Rm	CAATCGGAGTTCTTCGTGAT	qPCR primers cattle	Cattle *Bacteroides*	Mieszkin et al. (2010)

a Species that show the highest similarity to the probes according to BLAST analysis (http://blast.ncbi.nlm.nih.gov/Blast.cgi).

b The GC clamp attached to the 5′ end is denoted in italics.

The positive samples were reamplified by nested PCR using the pair of primers 32F‐GC and 580R (Table 1), and the PCR mixture prepared as described above. Conditions for the nested PCR were an initial denaturation step at 94°C for 5 min; then 35 cycles consisting of 94°C for 30 sec, 65°C for 1 min (this temperature was decreased by 1°C every cycle until the touchdown temperature of 63°C was reached), and 72°C for 1 min and 30 sec; and finally a 6 min extension at 72°C.

An aliquot of 5 *μ*L of each PCR or nested‐PCR product was analyzed by 1.5% agarose (*w*/*v*) gel electrophoresis. DNA bands were stained with ethidium bromide and visualized by fluorescing when exposed to UV light.

### DGGE analysis of PCR products

DGGE was performed with a DCode system (Bio‐Rad, Hercules, CA) as previously described (Ballesté and Blanch 2011). Electrophoresis was performed with 1‐mm thick 8% (*w/v*) polyacrylamide gels (30% acrylamide–bisacrylamide [37.5:1]) submerged in 1× Tris–acetate acid–EDTA (40 mmol/L Tris, 20 mmol/L sodium acetate, 1 mmol/L EDTA; pH = 7.4) at 60°C. About 800–1000 ng of nested‐PCR product from the environmental samples and 100 ng (NanoDrop ND‐1000 spectrophotometer; Thermo Scientific, Wilmington, DE) of nested‐PCR product from the reference strains were loaded into individual lanes in the gel. The following electrophoresis conditions were selected in accordance with the one of the perpendicular DGGE and time of travel experiments (data not shown): 17 h at 85 V and 60°C in a linear 40–65% denaturing gradient (100% denaturant agent was defined as 7 mol/L urea and 40% [*v/v*] formamide). The gels were stained for 45 min in 1X sodium chloride–Tris–EDTA buffer (100 mmol/L NaCl, 10 mmol/L Tris, 1 mmol/L EDTA; pH = 7.4) with SYBRGold nucleic acid stain (Molecular Probes Inc., Eugene, OR) and visualized under UV radiation using a ChemiDoc^™^ MP Imaging System (BioRad). The gels were scanned and analyzed using the Quantity One 4.6.7 program (Bio‐Rad).

### Extraction of DGGE bands and sequencing

DGGE bands that were present in all the samples with a particular fecal pollution source and absent in the rest were selected and excised with a sterile razor blade. They were introduced into the wells of 1.5% agarose gel, sealed with melted 1.5% agarose, and analyzed by electrophoresis. The bands were visualized via ethidium bromide staining. DNA was extracted and purified using a QIAquick^®^ gel extraction kit (Qiagen GmbH) following the manufacturer's instructions. The product was reamplified with the primers 32F and 580R (Table 1), and analyzed again by gel electrophoresis, and DNA was extracted and purified as explained above. The sequencing reaction was performed using a BigDye Terminator cycle‐sequencing ready‐reaction kit (Applied Biosystems) and by adding 5 *μ*L of DNA. The reaction was performed under the following conditions: 25 cycles of 96°C for 30 sec, 50°C for 5 sec, and 60°C for 4 min. The product was analyzed using an automated DNA sequencer (ABI Prism 3700; PerkinElmer, Thermo Fisher Scientific, Waltham, MA USA [service provided by the Serveis Cientificotècnics of the University of Barcelona]).

The 16S rRNA gene sequences were edited and aligned using version 7.0.1 of the BioEdit program (Hall 1999). BLAST (http://blast.ncbi.nlm.nih.gov/Blast.cgi) was used to search for homology of each sequence with *Bacteroides* spp. and, when the information was available, to look for coincidences with the corresponding fecal source.

### qPCR procedures

#### Clone construction

To generate standards for the qPCR assays, the four host‐specific 16S rRNA gene fragments were cloned in pGEM‐T Easy vectors following the manufacturer's instructions (Promega Biotech Ibérica, Barcelona, Spain). Each construction was transformed by electroporation (2.5 kV, 25 F capacitance, and 200 V resistance) into *E. coli* DH5*α* electrocompetent cells. The ampicillin‐resistant colonies that contained the vector with the insert were selected, verified by PCR, and plasmids were purified using a Qiagen Plasmid Midi purification kit (Qiagen Inc., Valencia, CA). A NanoDrop ND‐1000 spectrophotometer (Thermo Scientific) was used to evaluate the concentration and purity of the constructs containing each band.

To calculate the number of gene copies (GC) in the stock prepared for each gene, the following equation was used: [concentration of pGEM‐T Easy::*insert* (ng/*μ*L)/molecular mass (ng/mol)] × 6.022 × 10^23^ molecules/mol = number of molecules of pGEM‐T Easy::*insert*/*μ*L. Ten‐fold serial dilutions of the stock were performed with double‐distilled water and stored at −20°C until used. The stocks were amplified in triplicate in five independent experiments, and the average of the threshold cycle (C_t_) results was used to elaborate standard curves.

#### Bac‐Fw and Bac‐Rev primers and probe sets

The 16S rRNA gene sequences were edited and aligned using version 7.0.1 of the BioEdit program (Hall 1999). Primers and probes were selected in the sequence of each DGGE fragment of 16S rRNA genes using the software tool Primer Express 3.0 (Applied Biosystems) to be used in a standardized amplification protocol. A set of common forward and reverse primers and specific hydrolysis probes for each fecal source (human, pig, poultry, and cattle) were designed. In addition, a probe common to all the origins was also designed. All primers and hydrolysis probes were commercially synthesized by Applied Biosystems. HMprobeBac, PGprobeBac, PLprobeBac, CWprobeBac, and Bacprobe were MGB probes with an FAM reporter and a nonfluorescent quencher (NFQ). The amplification conditions were used as described previously (Gómez‐Doñate et al. 2012). Briefly, amplification was performed in a 20‐*μ*L reaction mixture with TaqMan Environmental Real‐Time PCR Master Mix 2.0 (Applied Biosystems). The mixture contained 900 nmol/L each primer, 250 nmol/L the corresponding probe, and 7 *μ*L of the DNA sample or quantified plasmid DNA. The thermal‐cycler conditions were as follows: an initial setup of 2 min at 50°C, followed by 10 min at 95°C, 40 cycles of 15 sec of denaturation at 95°C, and 1 min of annealing/extension at 60°C. All the samples, standards, and positive and negative controls were run in triplicate. The threshold cycle (C_t_) obtained was defined as the average of the triplicate data obtained. C_t_ data were expressed as the number of GC according to the values obtained with the standard for each qPCR reaction. In order to confirm the specificity of the primers and probes for their target genomes, NCBI (National Center for Biotechnology Information) data entries for *Bacteroides* spp. were used. Their specificity was also tested by cross reactions using DNA isolated from the other sources.

### Use of host‐specific MST markers based on *Bacteroidetes* qPCR methods

Three previously described qPCR assays used for MST studies were also tested for comparison with those developed in this study: HF183, specific for humans (Seurinck et al. 2005); Pig‐2‐Bac, specific for pigs (Mieszkin et al. 2009); and Rum‐2‐Bac, specific for ruminants (Mieszkin et al. 2010). The assays and conditions used were as previously reported. Briefly, HF183 is a real‐time polymerase chain reaction (PCR) assay using SybrR Green I (Table 1), while Pig‐2‐Bac and Rum‐2‐Bac are real‐time qPCR assays using FAM‐labeled TaqMan probes (Table 1). Wastewater samples from known and single fecal sources were used to test their specificities. Traditional indicators (*E. coli* and somatic coliphages) were enumerated as detailed in the section “Enumeration of *E. coli* and somatic coliphages” and used as references of the fecal load of the wastewater and slurry samples.

### Statistical analysis

Sensitivity (*r*), specificity (*s*), and accuracy (*a*) were calculated according to the following formulas: *r* = [TP/(TP + FN)]; *s* = [TN/(TN + FP)]; and *a* = [(TP + TN)/all samples], where TP is the number of samples that were positive for the qPCR marker of their own species (true positive), TN is the number of samples that were negative for a qPCR marker of another species (true negative), FP is the number of samples that were positive for a qPCR marker of another species (false positive), and FN is the number of samples that were negative for a qPCR marker of their own species (false negative).

### Theory

Host‐specific bacterial identification by DGGE pattern‐band technique was successfully applied in previous studies for identifying host‐specific *Bifidobacterium* (Ballesté and Blanch 2011). These strains were used to discriminate between human, cow, poultry, and pig fecal pollution from high and moderate polluted samples (Gómez‐Doñate et al. 2012). As *Bacteroides* spp. is one of the most abundant genera of bacteria in the gastrointestinal tract of humans and other warm‐blooded animals, the design of *Bacteroides* host‐specific qPCRs could become a robust and reliable technique for MST studies.

## Results

### DGGE analysis of PCR products

**Figure 1:**
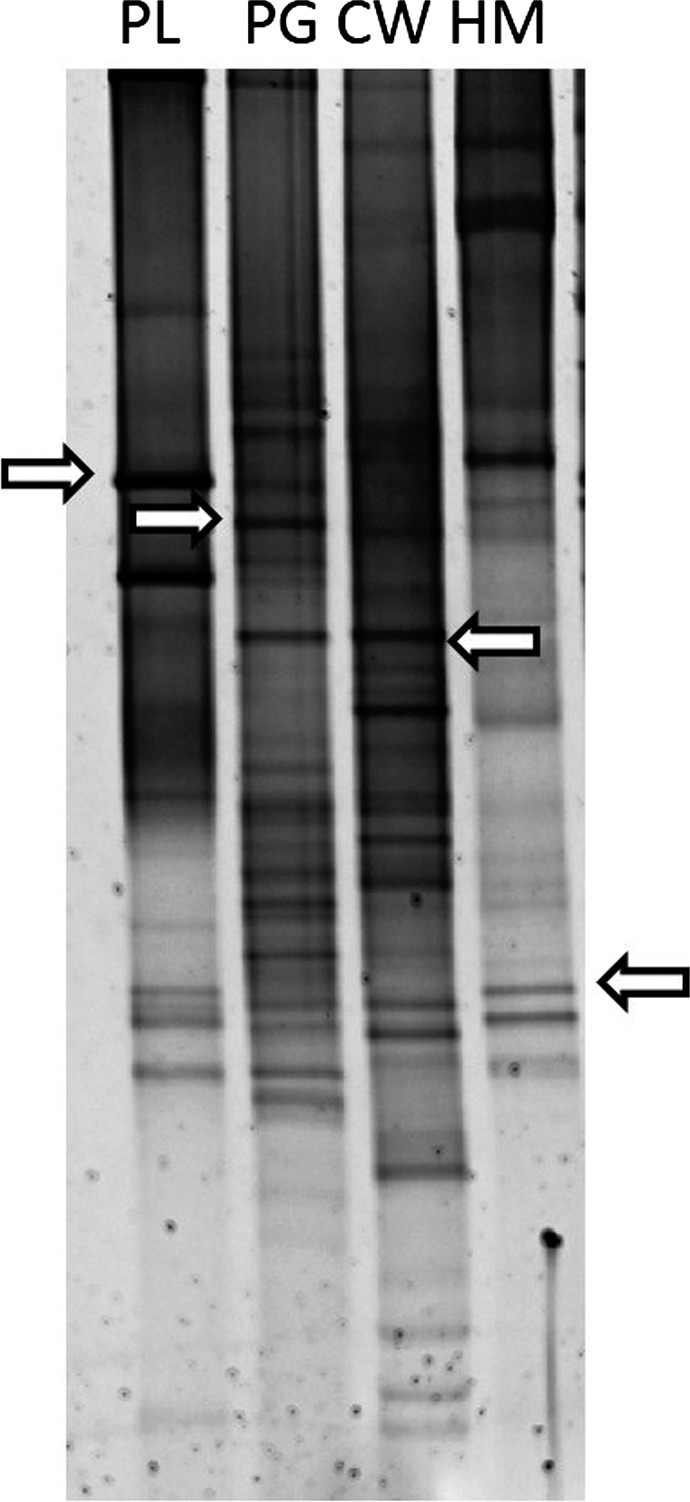
Representative denaturing gradient gel electrophoresis profiles of 16S rRNA of *Bacteroides* from wastewater samples of known fecal origin. Selected bands for each source are indicated with arrows (PL, poultry; PG, pig; CW, cattle; HM, human).

The DGGE profiles of the fragments corresponding to *Bacteroides* 16S rRNA genes varied depending on the source of the fecal pollution in the water sample. The profiles of the human and poultry wastewater samples showed fewer bands than the cow and pig samples (Fig. 1), which had more heterogeneous profiles. The broadest bands common to all the samples from the same source and differential from those of other fecal sources were selected for further analysis. The selected bands were sequenced and compared with the BLAST and NCBI (National Center for Biotechnology Information) databases. Finally, only one band for each fecal origin was selected (Fig. 1). The bands selected for each source were all identified by BLAST as *Bacteroides* spp.

### qPCR standards of host‐specific *Bacteroides* spp.

**Figure 2:**
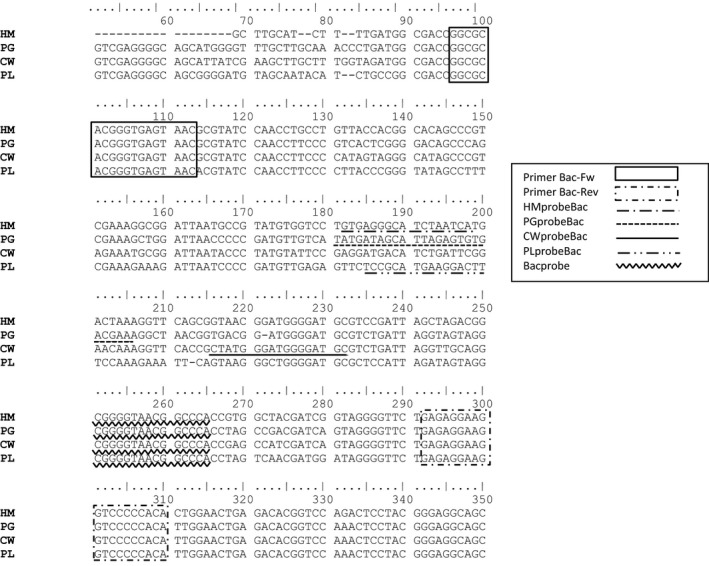
Alignment of the sequences of the 16S rRNA genes from isolated denaturing gradient gel electrophoresis bands of human (HM), pig (PG), cattle (CW), and poultry (PL) wastewater, and general for *Bacteroides* (Bac). The locations of the primers Bac‐FW and Bac‐Rev, and the set of probes (HMprobeBac, PGprobeBac, CWprobeBac, PLprobeBac and Bacprobe) are indicated.

A region of the 16S rRNA gene sequence which had a variable zone between two conserved areas for each of the four hosts was selected to design the forward and reverse primers, and the Bacprobe common to the four origins. The specific probes for each source of fecal pollution (human, cattle, poultry, and pig) and a probe common to the *Bacteroides* genus were designed in the variable zone of each respective sequence (Fig. 2).

Primers and probes were selected according to the requirements of the Primer Express 3.0 software (Applied Biosystems). Using the five qPCR assays, five standard curves were plotted: one for the assay targeting the *Bacteroides* genus regardless the fecal origin, and one assay specific to each of the four pollution sources. The fragment of the 16S rRNA gene amplified from each DGGE band and cloned to generate the standard curves had 500 bp for the human source, 541 bp for poultry, 478 bp for pig, 543 bp for cattle, and 543 bp for the common *Bacteroides* genus assay. By using the primers Bac‐FW and Bac‐Rev, the amplicon size for each fecal source was 215 bp for human, cattle, and the common *Bacteroides* genus probe; and 214 bp for poultry and pig. The standard curves were reproducible, and the average slopes of the three replicates of the five independent amplifications were −3.3725 with a standard deviation (SD) of 0.033 for humans; −3.5061 (SD, 0.076) for poultry; −3.4589 (SD, 0.079) for pig; −3.4900 (SD, 0.064) for cattle; and −3.2805 (SD, 0.023) for the *Bacteroides* genus. Accordingly, the amplification efficiencies (*E*) for the assays were as follows: 97.7% (range, 96.4–100.1%) for human origin; 92.9% (range, 90.1–98.9%) for poultry origin; 94.1% (range, 90.7–97.9%) for pig origin; 93.3% (range, 90.8–96.3%) for cattle origin; and 101.8% (range, 100.7–102.9%) for the *Bacteroides* genus. Since the number of copies of the 16S rRNA gene of different *Bacteroides* spp. are not yet well defined (Hong et al. 2008), the quantitative expression of the qPCR calculations was based on GC and not on the number of bacterial cells. Thus, the calculated limits of detection in the qPCR assay are: 21.1 GC/*μ*L for human; 13.9 GC/*μ*L for pig; 16.4 GC/*μ*L for poultry; 33.9 GC/*μ*L for cattle; and 33.7 GC/*μ*L for the *Bacteroides* genus.

### Performance of host‐specific qPCR assays with wastewater samples of known fecal origin

The new host‐specific *Bacteroides*‐based probes showed high specificity for their respective hosts (Tables 2, 3) in the 44 wastewater samples from the four origins, except for the cattle‐specific probe. Since the assay for cattle was not specific, it was only used to analyze five samples and dropped from further studies. The HMprobeBac and PLprobeBac qPCR assays (for human and poultry, respectively) did not show any false positives with the samples from other fecal sources, and the PGprobeBac qPCR assay (pig specific) showed false‐positive reaction with a cattle sample (Table 2).

**Table 2:** Microbial indicators and qPCR results for wastewater samples. Host‐specific *Bacteroides* qPCRs developed in this study: HMprobeBac (human), PLprobeBac (poultry), PGprobeBac (pig), CWprobeBac (cow), and Bacprobe (total *Bacteroides*). Host‐specific *Bacteroides* qPCRs developed by other authors and assayed in this study: HF183 (human), Pig‐2‐Bac (pig), and Rum‐2‐Bac (ruminants). SOMCPH: somatic coliphages. Relative abundance of *Bacteroides* cells in the samples were calculated with Bacprobe results considering five copies of 16S rRNA gene in *Bacteroides thetaiotaomicron* (Pei et al. 2010)

Source	Host‐specific *Bacteroides* qPCRs in this study (GC/mL)					Other proposed host‐specific targets (GC/mL)			*B. thetaiotaomicron*cells/mL	*Escherichia coli* (cfu/mL)	SOMCPH (pfu/mL)
	HMprobeBac	PLprobeBac	PGprobeBac	CWprobeBac	Bacprobe	HF183	Pig‐2‐Bac	Rum‐2‐Bac			
Human											
Positive (%)	5/11 (45.5)	0/11 (0)	0/11 (0)	5/5 (100)	11/11 (100)	6/6 (100)	2/9 (22.2)	0/11 (0)		11/11 (100)	11/11 (100)
Max value	2.05 × 10^9^	0	0	4.59 × 10^6^	1.80 × 10^8^	1.14 × 10^7^	2.12 × 10^6^	0	3.6 × 10^7^	9.15 × 10^6^	9.77 × 10^5^
Min value	0	0	0	4.44 × 10^4^	4.56 × 10^4^	4.42 × 10^6^	0	0	9.1 × 10^3^	2.01 × 10^4^	8.80 × 10^3^
Mean^a^	7.95 × 10^7^			1.48 × 10^6^	1.28 × 10^7^	7.56 × 10^6^	2.12 × 10^5^		2.6 × 10^6^	2.96 × 10^5^	4.20 × 10^4^
Poultry											
Positive (%)	0/11 (0)	8/11 (72.7)	0/11 (0)	5/5 (100)	11/11 (100)	6/6 (100)	5/9 (55.6)	0/11 (0)		11/11 (100)	11/11 (100)
Max value	0	2.84 × 10^9^	0	2.52 × 10^6^	1.28 × 10^9^	3.61 × 10^6^	2.99 × 10^6^	0	2.6 × 10^8^	1.72 × 10^5^	9.25 × 10^6^
Min value	0	0	0	5.74 × 10^3^	6.25 × 10^5^	2.03 × 10^6^	0	0	1.2 × 10^5^	6.31 × 10^4^	2.70 × 10^2^
Mean^a^		5.42 × 10^7^		1.61 × 10^5^	3.93 × 10^7^	2.82 × 10^6^	6.71 × 10^5^		7.9 × 10^6^	2.70 × 10^5^	4.20 × 10^4^
Pig											
Positive (%)	0/12 (0)	0/12 (0)	6/12 (50)	5/5 (100)	12/12 (100)	5/9 (55.6)	12/12 (100)	0/12 (0)		12/12 (100)	12/12 (100)
Max value	0	0	5.68 × 10^7^	5.52 × 10^8^	5.24 × 10^8^	9.42 × 10^5^	3.03 × 10^8^	0	1.1 × 10^8^	6.00 × 10^5^	2.95 × 10^5^
Min value	0	0	0	5.98 × 10^5^	2.39 × 10^5^	0	1.10 × 10^7^	0	4.8 × 10^4^	1.35 × 10^4^	1.57 × 10^4^
Mean^a^			5.40 × 10^6^	2.80 × 10^7^	5.61 × 10^7^	1.23 × 10^5^	4.15 × 10^7^		1.1 × 10^7^	7.80 × 10^4^	5.65 × 10^4^
Cattle											
Positive (%)	0/10 (0)	0/10 (0)	1/10 (10)	5/5 (100)	10/10 (100)	5/5 (100)	7/10 (70)	7/10 (70)		10/10 (100)	10/10 (100)
Max value	0	0	4.86 × 10^2^	1.16 × 10^7^	1.80 × 10^9^	3.32 × 10^6^	3.45 × 10^6^	2.70 × 10^3^	3.6 × 10^8^	3.54 × 10^6^	3.25 × 10^6^
Min value	0	0	0	2.97 × 10^5^	5.79 × 10^6^	1.04 × 10^5^	0	0	1.2 × 10^6^	2.51 × 10^4^	1.00
Mean^a^			4.86 × 10^2^	2.28 × 10^6^	1.46 × 10^8^	4.12 × 10^5^	2.02 × 10^4^	2.06 × 10^2^	2.9 × 10^7^	2.14 × 10^5^	4.73 × 10^3^

a Geometric mean calculated from positive results.

**Table 3:** Sensitivity, specificity, and accuracy of the qPCR assays analyzed. Host‐specific *Bacteroides* qPCRs developed in this study: HMprobeBac (human), PLprobeBac (poultry), PGprobeBac (pig), CWprobeBac (cow), and Bacprobe (total *Bacteroides*). Host‐specific *Bacteroides* qPCRs developed by other authors and assayed in this study: HF183 (human), Pig‐2‐Bac (pig), and Rum‐2‐Bac (ruminants)

Value	Host‐specific *Bacteroides* qPCRs in this study					Other proposed host‐specific targets		
	HMprobeBac	PLprobeBac	PGprobeBac	CWprobeBac	Bacprobe	HF183	Pig‐2‐Bac	Rum‐2‐Bac
Sensitivity	0.45	0.73	0.50	1.00	1.00	1.00	1.00	0.70
Specificity	1.00	1.00	0.97	0.00	1.00	0.20	0.50	1.00
Accuracy	0.86	0.93	0.84	0.25	1.00	0.38	0.65	0.93

The common qPCR assay for the *Bacteroides* genus showed amplification in all the samples tested. The concentration detected was equal to or greater than that of the corresponding host‐specific *Bacteroides* qPCR assay (Table 2). This observation suggests that the qPCR assay for the *Bacteroides* genus not only detects the studied fecal sources present in the sample, but also other *Bacteroides* strains that are part of host's gastrointestinal microbiota present in the samples.

Host‐specific *Bacteroides* qPCRs assays previously described in other studies were incorporated to our study with the same samples. Because these assays were later incorporated to the study, with some assays less samples were analyzed (Table 2). The human specific qPCR HF183 (Seurinck et al. 2005) showed 100% detection with human samples but 88% of false positives with samples of different fecal origins. The pig‐specific qPCR assay Pig‐2‐Bac (Mieszkin et al. 2009) showed 100% amplification of pig samples, but 50% false positives, especially with poultry and cattle samples. Finally, the cattle qPCR Rum‐2‐Bac assay (Mieszkin et al. 2010) was 100% specific (Table 3), without any cross reactivity, with 70% of cattle samples showing positive amplification, although the GC detected was lower than for the other host‐specific qPCR assays.

Thus, the host‐specific *Bacteroides* qPCRs designed in this study for human and pig showed a higher specificity in samples from Northeastern Spain than the previously designed ones (HF183 and Pig‐2‐Bac) for the Bacteroidetes group. In contrast, the Rum‐2‐Bac outperformed our cattle‐specific assay (CWprobeBac) (Table 3).

## Discussion

*Bacteroides* spp. have been proposed as indicators of fecal pollution in water because they fulfill most of the requirements of a fecal indicator (Fiksdal et al. 1985; USEPA 2005; Hurst 2007). Moreover, it has been reported that there are some species of *Bacteroides* which are highly host‐specific, and which can be used to discriminate the sources of fecal pollution in water (Kreader 1995; Harwood et al. 2014). *Bacteroides* is an strictly anaerobic bacteria and hence not able to survive under oxygen stress conditions (Avelar et al. 1998). The reduced environmental persistence of this genus makes its detection by cultivable methods difficult. It is therefore advisable to target the corresponding 16S rRNA gene using culture‐independent molecular methods (Kreader 1998; Ballesté and Blanch 2010).

In this study, 16S rRNA gene amplification with specific primers for *Bacteroides* genus, followed by DGGE analysis, allowed us to differentiate bands with different sequences belonging to different *Bacteroides* spp. for each fecal origin. It has been considered that, based on the 16S rRNA gene sequences, DGGE is sufficiently sensitive to allow detection of bacteria that constitute up to 1% of the total bacteria community (Muyzer and Smalla 1998). Thus, in our study, only the most predominant *Bacteroides* spp. should have been detected. In some cases, however, different DNA sequences present the same melting domains (Ballesté and Blanch 2011), as observed with bands selected for pig and cow (Fig. 1).

Using this combined 16S rRNA gene amplification and DGGE technique, it was possible to observe that human and poultry samples showed less diversity in the 16S rRNA fragments within their *Bacteroides* populations than the pig and cattle samples. The most useful band for each fecal origin was selected on the basis of the most dominant band coincident in various samples analyzed of the same origin and its absence in samples of other origins. There is a delicate equilibrium between the required specificity and the need to cover most *Bacteroides* spp. within the same fecal source. The problem of samples showing negative results within their own source could be due to the presence of an abundant *Bacteroides* spp. population with a 16S rRNA gene showing a different sequence than that of the band selected in our study. Despite 114 samples being used for the selection of the most useful band, the analysis of more samples or samples from different sites would allow the evaluation of the extent of the false‐negative results, considering the high diversity observed. For example, some poultry samples were collected from abattoirs that sacrifice different animal species. Since each organism has specific *Bacteroides* microbiota, which has coevolved with it, it is possible that the band selected belonged, for instance, to the *Bacteroides* species corresponding to hens and therefore there would be no amplification if a sample contained fecal pollution from turkey. The variability within the Bacteroidales group has previously been reported and it has been suggested that multiple targets could be necessary to accurately assess fecal pollution (Lamendella et al. 2013).

Meanwhile, the lack of specificity observed in the assay intended to be specific for cattle could be attributable to the opposite effect. In addition to strains in cattle, the sequence could also be present (albeit in minimal proportions, hence not visible by DGGE) in other sources. This limitation may be overcome by increasing the number of samples analyzed by DGGE and the selection of a new band; although our attempts have resulted unsuccessful so far (data not shown).

Concerning the common *Bacteroides* genus assay, it showed detection in all the fecal samples, showing GC equal to or higher than the respective specific assay. This observation could be explained by this assay not only detecting a specific target linked with a fecal origin, as the specific assays do, but also most of the *Bacteroides* strains present in the gastrointestinal tract regardless the host.

The problems encountered with the qPCR assays designed in this study appear to be shared with other assays. When using the host‐specific Bacteroidetes assay (Seurinck et al. 2005; Mieszkin et al. 2009), it was observed that the human HF183 and pig Pig‐2‐Bac assays showed good sensitivity but a lower specificity than the host‐specific *Bacteroides* qPCR assays developed in this study. It should be considered that these assays were designed from samples from different geographical regions. Differences in the *Bacteroides* strains, as well as in the bacteriophages infecting *Bacteroides*, have been observed in samples with different geographical origins (Puig et al. 1999; Ishikawa et al. 2013; Lin et al. 2013). The reason for these differences is not clear, but they could be due to different selection of gut microbiota in different ethnic groups or the influence of different feeding habits (Yatsunenko et al. 2012; Kelder et al. 2014), and because *Bacteroides* spp. have established close mutualism with their hosts (Bäckhed et al. 2005). Finally, the cow‐specific assay, Rum‐2‐Bac, showed a high specificity for its fecal source. Unfortunately, in our samples, we detected a low GC concentration (2 logarithmic units) in highly polluted samples. These low concentrations may be limiting the use of Rum‐2‐Bac assay in environmental samples with a low fecal pollution level. Overall, the host‐specific *Bacteroides sp*. qPCRs designed in this study for human (HMprobeBac) and pig (PGprobeBac) presented a higher accuracy than the HF183 and Pig‐2‐Bac qPCRs, respectively.

Certain limitations of all *Bacteroides* qPCR methods should be considered when selecting the specific assay. First, the sensitivity of the assay if it is intended to apply it in MST in environments that present dilute or aged fecal pollution. Second, the selection of the method should take into account the area where it is to be applied. Validation of a given assay with local samples could reveal different outcomes from the results reported with samples from the location where the method was developed.

## Conclusion

DGGE is a useful technique for identifying host‐specific bacteria from single fecal polluted samples. MST molecular targets based on these host‐specific *Bacteroides* allowed the design of host‐specific qPCR methods, with the exception of cow‐specific one. Although qPCR molecular targets showed moderate sensitivity (Jofre and Blanch 2010), these particular MST molecular indicators were rapid, straightforward, and accurate. These methods could be applied, together with traditional microbial indicators, for the development of predictive models, as previously reported (Ballesté and Blanch 2010; Sánchez et al. 2011).

## Conflict of Interest

None declared.
